# Supplementary material for: Association between oral microbiome and seven types of cancers in East Asian population: a two-sample Mendelian randomization analysis
Source: Front Mol Biosci. 2023 Nov 21;10:1327893. doi: 10.3389/fmolb.2023.1327893 (PMC10702768; doi:10.3389/fmolb.2023.1327893)
Supplement: Supplementary file 1 [file DataSheet1.ZIP › Supplementary Materials/MR plots of saliva/saliva═╝/Lung Cancer/pheno.388_to_lung cancer_leave_one_out.pdf]

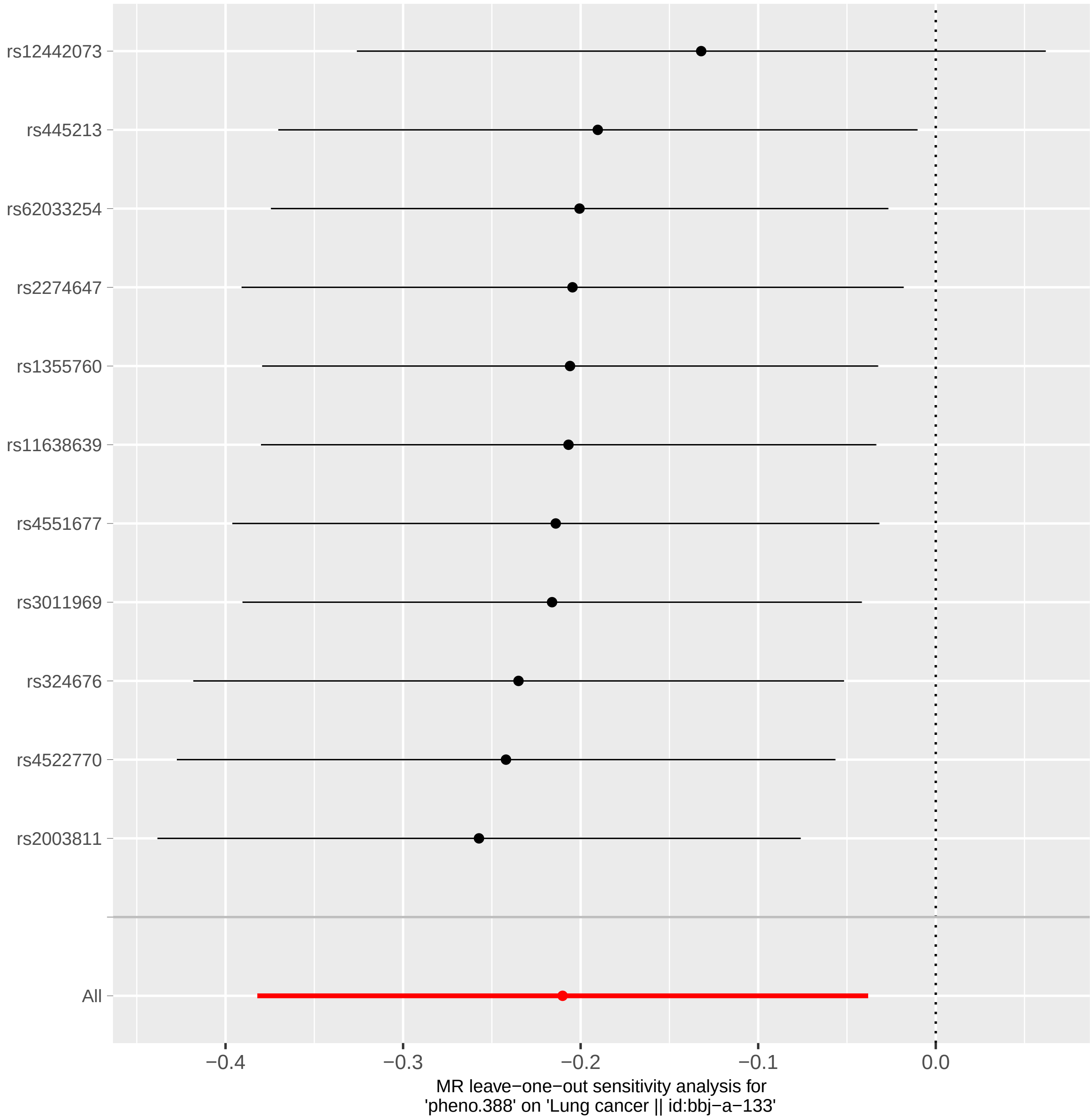
rs12442073
rs445213
rs62033254
rs2274647
rs1355760
rs11638639
rs4551677
rs3011969
rs324676
rs4522770
rs2003811
All
−0.4
−0.3
−0.2
−0.1
0.0
MR leave−one−out sensitivity analysis for
'pheno.388' on 'Lung cancer || id:bbj−a−133'
